# The nociceptin/orphanin FQ receptor agonist SR-8993 as a candidate therapeutic for alcohol use disorders: validation in rat models

**DOI:** 10.1007/s00213-016-4385-8

**Published:** 2016-08-11

**Authors:** Abdul Maruf Asif Aziz, Shaun Brothers, Gregory Sartor, Lovisa Holm, Markus Heilig, Claes Wahlestedt, Annika Thorsell

**Affiliations:** 1 Center for Social and Affective Neuroscience, Department of Clinical and Experimental Medicine (IKE), Linköping University, SE-581 83 Linköping, Sweden; 2 University of Miami Health System, University of Miami, Miami, FL USA

**Keywords:** Nociception/orphanin FQ, Agonist, Wistar rat, Alcohol, Operant, Reinstatement, Elevated plus-maze

## Abstract

**Abstract:**

**Rationale:**

Alcoholism is a complex disorder in which diverse pathophysiological processes contribute to initiation and progression, resulting in a high degree of heterogeneity among patients. Few pharmacotherapies are presently available, and patient responses to these are variable. The nociceptin/orphanin FQ (NOP) receptor has been suggested to play a role both in alcohol reward and in negatively reinforced alcohol seeking. Previous studies have shown that NOP-receptor activation reduces alcohol intake in genetically selected alcohol-preferring as well as alcohol-dependent rats. NOP activation also blocks stress- and cue-induced reinstatement of alcohol-seeking behavior.

**Objectives:**

Here, we aimed to examine a novel, potent, and brain-penetrant small-molecule NOP-receptor agonist, SR-8993, in animal models of alcohol- as well as anxiety-related behavior using male Wistar rats.

**Results:**

SR-8993 was mildly anxiolytic when given to naïve animals and potently reversed acute alcohol withdrawal-induced (“hangover”) anxiety. SR-8993 reduced both home-cage limited access drinking, operant responding for alcohol, and escalation induced through prolonged intermittent access to alcohol. SR-8993 further attenuated stress- as well as cue-induced relapse to alcohol seeking. For the effective dose (1.0 mg/kg), non-specific effects such as sedation may be limited, since a range of control behaviors were unaffected, and this dose did not interact with alcohol elimination.

**Conclusion:**

These findings provide further support for NOP-receptor agonism as a promising candidate treatment for alcoholism and establish SR-8993 or related molecules as suitable for further development as therapeutics.

## Introduction

Nociceptin/orphanin FQ (N/OFQ), a heptadecapeptide, was discovered as an endogenous ligand for the orphan G-protein-coupled receptor opioid receptor like-1 (ORL1), or the nociceptin/orphanin FQ (NOP; (Meunier 1997; Meunier et al. 1995; Reinscheid et al. 1995)) receptor. NOP has since been identified as the fourth member of the opioid receptor family (Bunzow et al. 1994). Although the NOP receptor shares significant sequence homology with classical opioid receptors, N/OFQ does not bind to mu-, delta-, and or kappa-opioid receptors. Within the central nervous system, NOP is widely expressed in areas involved in alcohol reward, as well as those mediating negative affective responses and negatively reinforced alcohol seeking, such as the substantia nigra, hippocampus, dorsal raphe, amygdala, the hypothalamus, the bed nucleus of the stria terminalis, and the periaqueductal gray (Eckardt et al. 1998; Koob and Volkow 2010; Neal et al. 1999a; Neal et al. 1999b; Reinscheid et al. 2000; Wise 1998).

Evidence suggests that the N/OFQ–NOP system is a promising target for pharmacotherapy aimed at reducing excessive alcohol consumption and preventing relapse. Studies in genetically selected Marchigian Sardinian alcohol-preferring (msP) rats suggest that impaired function of this system may be an innate factor in high alcohol intake. Administration of N/OFQ reduces alcohol intake in the msP line while being ineffective in non-selected Wistar rats (Ciccocioppo et al. 2004; Ciccocioppo et al. 1999). An impairment of NOP-receptor G-protein coupling is present in central amygdala (CeA) of mSP rats and can be overcome by intracerebroventricular or CeA injections of N/OFQ to rescue the excessive alcohol consumption of this line (Ciccocioppo et al. 2003; Economidou et al. 2008). Furthermore, neuroadaptations within the N/OFQ–NOP system occurring as a result of alcohol dependence may similarly be involved in escalation of alcohol consumption. Wistar rats without a history of dependence are not sensitive to suppression of alcohol self-administration or stress responses by N/OFQ. However, a history of dependence results in escalation of alcohol self-administration, sensitized stress responses, and increased expression of NOP. Under these conditions, administration of N/OFQ reduces alcohol intake and produces anxiolytic-like effects (Martin-Fardon et al. 2010). Additionally, preclinical studies suggest a potential of NOP agonists to prevent relapse, with a broader than usual activity profile. Most experimental therapeutics blocking reinstatement of alcohol seeking do so either when this behavior is triggered by stress- or alcohol-associated cues. In contrast, central administration of N/OFQ suppresses relapse-like behavior induced by both these classes of stimuli (Ciccocioppo et al. 2004; Martin-Fardon et al. 2000).

The first small-molecule, brain-penetrant NOP-receptor agonist that was disclosed was Ro 64-6198. It showed >100-fold selectivity for NOP over classical opioid receptors and produced anxiolytic-like effects in vivo (Jenck et al. 2000; Wichmann et al. 2000). Ro 64-6198 was subsequently reported to block acquisition, expression, and reinstatement of alcohol-conditioned place preference in mice (Kuzmin et al. 2003). Additionally, it decreased operant alcohol self-administration, as well as relapse-like drinking in Wistar rats. However, when given to the msP rat, Ro 64-6198 increased rather than decreased alcohol consumption (Economidou et al. 2006; Kuzmin et al. 2007). These seemingly contradictory results may be due to residual activity of Ro 64-6198 on the mu-opioid receptor (Dautzenberg et al. 2001). Another brain-penetrant small-molecule NOP agonist with sub-nanomolar affinity for the receptor and largely devoid of mu-opioid activity, W-212393, was subsequently disclosed by Teshima and colleagues and reported to influence circadian body-temperature variation in rats (Teshima et al. 2005). The hydrochloride of W-212393, MT-7716, was then evaluated for its activity in alcohol models (Ciccocioppo et al. 2014). MT-7716 given orally for 14 days dose-dependently decreased voluntary alcohol intake in msP rats, an effect that remained 1 week after discontinuation of the drug. Similar to N/OFQ itself, MT-7716 also prevented reinstatement caused by both alcohol-associated stimuli and stress. Finally, it attenuated somatic symptoms of alcohol withdrawal.

Despite these findings, no NOP agonist has to our knowledge reached clinical development for alcohol use disorders. N/OFQ and synthetic NOP agonists present to date are sedative when given at high doses. Therefore, insufficient separation between specific effects and sedative actions may have posed a challenge for drug development efforts targeting this mechanism. A need remains to develop NOP agonists with potential to be developed into therapeutics. The present study presents a novel, small-molecule NOP agonist, SR-8993. SR-8993 is a brain penetrant upon peripheral administration (intraperitoneal; i.p.) and achieves pharmacologically active central concentrations following intraperitoneal administration. Additionally, it has >100-fold selectivity for the NOP-receptor over the μ-opioid receptor and essentially no activity at the κ- or δ-opioid receptor (Andero et al. 2013). Here, we evaluated its activity in alcohol-related behaviors. We assessed withdrawal-induced anxiety-like behavior, free-choice alcohol drinking, operant alcohol self-administration, withdrawal-induced anxiety, and relapse-like behavior. We also examined the potential role of prior alcohol dependence for the activity of SR-8993. Finally, we evaluated the behavioral specificity of effects observed in the alcohol models, as well as potential pharmacokinetic SR-8993—alcohol interactions.

## Materials and methods

### Animals

Male Wistar rats (250–300 g at start of experiments; Charles River, Horsham, PA, USA (NIAAA, NIH); Taconic, Ejby, Denmark (Linkoping University, Linkoping)) were used throughout. Animals were group-housed two-four per cage in a temperature- and humidity-controlled vivarium, using a reverse 12 h light/dark cycle, lights off at 8:00 A.M. Food and water were available ad libitum, except during the initial operant training. Experiments were conducted in accordance with the National Institutes of Health Guide for the Care and Use of Laboratory Animals (work done at the NIAAA, NIH) or the European Union guidelines on the care and use of laboratory animals. All experimental procedures were approved by the local ethics committee on animal research (NIAAA ACUC and the Research Animal Ethics Board in Linköping).

### Drug

The NOP-receptor agonist, SR-8993 (see (Andero et al. 2013) for structural information as well as selectivity) was developed, synthesized, and provided by the University of Miami, Florida, USA. The drug was dissolved in 10 % DMSO, 10 % Tween-80 and sterile water. All injections were made i.p. 45 min prior to testing.

### Elevated plus-maze

Anxiety-like behavior was evaluated in the elevated plus-maze (EPM) as previously described, e.g., (Thorsell et al. 2007) with testing under red light. The rat was placed on the central platform facing an open arm and behavior recorded for 5 min using an automated tracking system (EthoVision, Noldus, Wageningen, Netherlands). The maze was cleaned with 10 % alcohol solution between the tests. The anxiety-related outcome measures used for analysis were %open time and %open entries, defined as 100 * open/(open + closed). The total number of closed arm entries was used as an index of activity.

### Acute alcohol withdrawal (“hangover”) anxiety

Alcohol-induced withdrawal anxiety was measured using the EPM with a preceding acute administration of alcohol (3 g/kg; 12 h prior to EPM) as previously described (Gehlert et al. 2007). Animals were treated with vehicle or SR-8993 (1.0 mg/kg).

### Open field

The open field consisted of an automated setup using infra-red beams to detect activity (field size, 43 × 43 cm; Med Associates Inc., Georgia, VT, USA). The rat was placed into the center of the field, and testing was done under dim white light (about 50 lx). Animals were allowed 10 min habituation on the day preceding testing to eliminate the influence of novelty on general locomotion. Drug effects were tested for 15 min (vehicle, 1 mg/kg or 3 mg/kg SR-8993, *n* = 8/group). The open-field arena was cleaned with 10 % alcohol solution and dried between subjects.

### Forced swim test

Antidepressant-like effects were examined in the forced swim test (FST) (Thorsell et al. 2007). The apparatus used was an 80-cm-high cylinder (diameter appr. 30 cm) filled to about two thirds with water (temperature 23-25 °C; changed after every three rats). On the pre-test day, animals were put in the water for 10 min. Following this session, the animal was removed from the water, dried off with a towel, and maintained in a cage placed half-on/half-off a warm blanket until fully dry at which time they were returned to their home cage. The following day, the drug effects were tested during a 5-min session (vehicle or 1 mg/kg SR-8993, *n* = 8/group). Measures scored were the following: latency to become immobile after being placed in the apparatus and the proportion of time spent immobile.

### Alcohol self-administration

Effects on oral operant self-administration of 10 % ethanol in water following saccharin fading were examined (Cippitelli et al. 2010). Testing was done after establishing a stable baseline. Animals (*n* = 11–12 per group) were treated with SR-8993 (0.3, 1, or 3 mg/kg) or vehicle. A Latin square, within-subject design was used with a minimum of 2 days of self-administration recovery between test days.

### Progressive-ratio responding

Rats (*n* = 8 per group) were trained to self-administer 10 % alcohol in the same manner as for alcohol self-administration testing. When a stable baseline of active lever pressing was reached (15 days), rats were treated (SR-8993 (1 mg/kg) or vehicle) and tested. The number of lever presses required to receive alcohol during this PR test (breakpoint) was 1, 2, 3, 4, 6, 8, 10, 12, and 16, after which the breakpoint continued to increase by four until the end of the test session (2 h; (Cippitelli et al. 2008)). Rats were determined to have reached their breakpoint when 30 min had passed before they pressed the active lever enough times to reach the next level.

### Cue- and stress-induced reinstatement

Reinstatement experiments were carried out as described (Cippitelli et al. 2010). For cue-induced reinstatement, rats (*n* = 10–12 per group) were presented with an orange cue scent during the sessions in which 10 % alcohol was given. After a stable baseline of lever-responding was reached, animals went through an extinction period during which alcohol and cues were absent. Once the extinction criterion (<10 lever presses per 30-min session) was reached, animals were randomly assigned to either vehicle or 1-mg/kg SR-8993 treatment group. For stress-induced reinstatement, animals (*n* = 12 per group) received yohimbine (1.25 mg/kg, i.p.). Thirty minutes following yohimbine treatment, the reinstatement test was started under the same conditions as extinction sessions. Responding on the inactive lever was recorded throughout all the experiment to monitor possible non-specific behavioral effects.

### Saccharin self-administration

Self-administration of 0.1 % saccharin was performed under the same conditions as alcohol self-administration (FR-1, TO 5s; 0.1-ml reward). Following establishment of a stable baseline, animals were randomized to treatment groups (vehicle or SR-8993 at 1 mg/kg) and responding for saccharin tested.

### Two-bottle free-choice drinking (4-h limited access)

Alcohol intake was determined using a limited access, two-bottle choice method. Animals were single caged, and alcohol made available in increasing concentration (3, 6 and 9 %) with 0.1 % saccharin. A second bottle containing only 0.1 % saccharin provided a free-choice situation. The position of the bottles was changed every day to prevent place preference. After establishing a stable intake baseline, rats were treated with SR-8993 (1 mg/kg) or vehicle (*n* = 8 per group), and intake was measured at 90 min and 4 h. The animals were weighed in order to calculate intake of alcohol per unit bodyweight. Alcohol intake (gram/kilogram) was calculated as well as alcohol preference (% ml alcohol solution/total volume (alcohol + water)).

### Intermittent access to 20 % alcohol (two-bottle choice procedure)

Escalation of alcohol consumption was achieved by intermittent access to 20 % alcohol (Simms et al. 2008). Animals were single housed with access to alcohol during 3 × 24 h per week (Sunday, Tuesday, and Thursday). Concurrent access to water was given and placement of the bottles alternated between sessions. Following exposure for 15 weeks, SR-8993 (0.3, 1, and 3 mg/kg) was administered and alcohol and water intake recorded 1, 2, 4, and 24 h following drug administration. Bodyweights were recorded on the day of treatment and the day after. Alcohol intake was calculated in grams/kilogram bodyweight. Alcohol preference was expressed as volume-percentage.

### Twenty percent *v*/*v* alcohol self-administration following intermittent access-induced escalation

Training and testing were conducted in the operant conditioning chambers described above but without saccharin fading. As opposed to alcohol solutions of lower concentrations, rats readily self-administer 20 % alcohol without saccharin fading. Rats were trained to self-administer 20 % alcohol (*v*/*v*) in 30-min daily sessions on a fixed ratio 1 (FR-1). Baseline training was for 3 weeks following which the rats were treated (1 mg/kg SR-8993 or vehicle) in a between-subject design.

Because animals used in the 20 % alcohol self-administration experiment had previously been exposed to treatment with SR-8993 during intermittent access, they were re-randomized to treatment group for the self-administration experiment. Self-administration data were then analyzed using a nested design, which evaluated a possible influence of prior treatment history. Washout time between the two experiments was 5 weeks.

### Taste preference

Taste preference was tested (Goodwin and Amit 1998) using a two-bottle free-choice procedure with continuous access to water and water with the addition of 0.2 % saccharin and 0.001 % quinine. Following stable consumption of respective solution, animals were treated with vehicle or SR-8993 (1 mg/kg) and cumulative liquid consumption was recorded for 24 h.

### Loss of righting reflex

An alcohol dose of 3.0 g/kg (appr. 20 ml/kg bodyweight) of 20 % (*v*/*v*) alcohol was administered i.p. to animals (*n* = 16) 45 min after treatment with SR-8993 (1 mg/kg) or vehicle. Rats were then placed in a supine position upon loss of consciousness, and the time was measured until the animals regained their righting reflex (having the ability to turn themselves over into the upright position after being in the supine position three times within 1 min). Blood alcohol content (BAC) upon waking was also determined.

### Blood alcohol concentration

The Ethanol Assay Kit from Sigma-Aldrich (Stockholm, Sweden) was used according to manufacturer’s instructions to determine BAC following the loss of righting reflex (LORR).

### Statistics

Behavioral data were evaluated using the GraphPad Prizm (v. 5.04; GraphPad Software, Inc., La Jolla, CA, USA) and SPSS (v. 22; IBM, Stockholm, Sweden). One-way repeated-measure ANOVA was used to analyze the self-administration data with treatment as factor. Cue-induced reinstatement data were analyzed using a two-way ANOVA (with session as the within-subjects factor and treatment as the between-subjects factor), while the stress-induced reinstatement data were analyzed with a two-way repeated-measure ANOVA (with session as the within-subjects factor and treatment as the between-subjects factor). Self-administration testing following intermittent access to 20 % alcohol was analyzed for a carry-over effect from treatment during intermittent access using a nested design in order to account for the treatment group during intermittent access. No effect of the intermittent access group on subsequent responding in the operant self-administration could be seen; therefore, analyses of baseline vs. escalated intake (“vehicle”) and treatment effect were analyzed separately using unpaired *t* tests. Treatment effect in the intermittent access paradigm was analyzed at the 24-h timepoint using a one-way ANOVA. Locomotor activity was analyzed using a one-way ANOVA. Unpaired *t* tests were used for the progressive ratio and loss of righting reflex data. The Newman–Keul test was used for all post hoc analyses.

## Results

### SR-8993 reverses acute alcohol withdrawal-induced anxiety in the elevated plus-maze (Fig. 1)

**Fig. 1:**
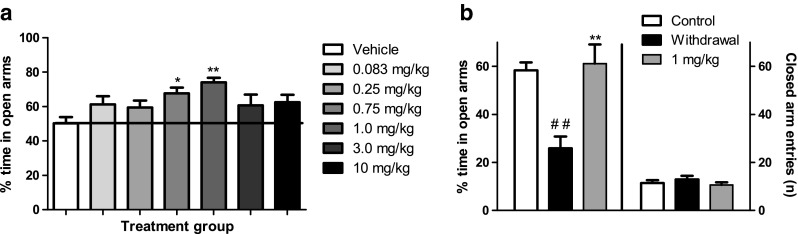
Behavioral screening for effective dose using the elevated plus-maze (**a**) and testing for reversal of alcohol-induced hangover anxiety (**b**). **a** Pre-treatment with SR-8993 at a dose of 1 mg/kg bodyweight had a pronounced anxiolytic-like effect on the elevated plus-maze during baseline, non-stressful conditions. Data presented is percent time spent on the open arm out of time spent on any arm (100 % × (open/(open + closed)). **b** The same dose was effective in reversing the anxiogenic effects of an acute administration of a high dose of alcohol. Withdrawal anxiety was induced by an acute administration of alcohol (i.p., 3.5 g/kg) at 12 h prior to testing using the elevated plus-maze, and the pre-treatment with SR 8993 (1 mg/kg) was administered 45 min prior to plus-maze exposure. Data presented is percent time spent on the open arm out of time spent on any arm (100 % × (open/(open + closed)) as well as the number of closed arm entries, a measure of locomotor activity. All values are given as mean ± SEM. **a** * *p* < 0.05 vs. vehicle, ** *p* < 0.01 vs. vehicle. **b** * *p* < 0.05 vs. vehicle, ## *p* < 0.01 vs. non-alcohol control, ** *p* = 0.01 vs. withdrawal

In the elevated plus-maze during non-challenged conditions, SR-8993 had a modest but statistically significant anxiolytic-like effect at intermediate doses (0.75–1.0 mg/kg; main effect of treatment, F[6, 80] = 3.104, *p* < 0.01; 0.75 mg/kg *p* < 0.05; 1.0 mg/kg *p* < 0.01 vs. vehicle; Fig. 1a).

One milligram per kilogram of SR-8993 fully reversed the anxiogenic-like effect observed 12 h following alcohol-administration (F[2,33] = 6.03, *p* < 0.001; post hoc, saline vs. alcohol *p* < 0.001; alcohol vs. SR-8993 1 mg/kg *p* < 0.001; Fig. 1b).

### SR-8993 reduces alcohol intake in two-bottle free-choice limited access (Fig. 2)

**Fig. 2:**
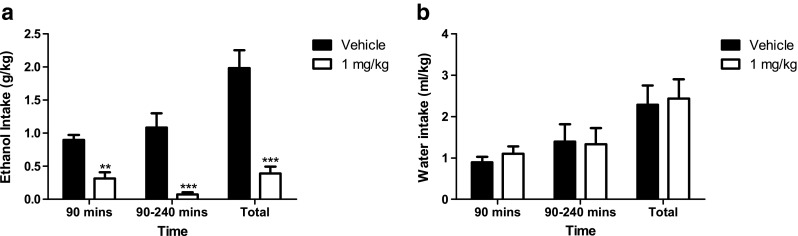
Alcohol intake (**a**) but not water intake (**b**) in a non-operant, two-bottle choice—paradigm was significantly attenuated by pre-treatment with SR-8993. Animals were given access to two bottles, one with alcohol solution and one with water, for 4 h starting 1 h into the dark phase. Measures are shown as intake at 90 min, intake between minute 90 and minute 240, and total intake during the session. **a** Alcohol intake was significantly suppressed at all timepoints examined. **b** Water intake (ml/kg bodyweight) was not affected by pre-treatment with SR-8993 at any timepoint. All values are given as mean ± SEM. ** *p* < 0.01 vs. vehicle, *** *p* < 0.001 vs. vehicle

Two-bottle choice drinking (limited 4-h access) was significantly reduced by SR-8993 (1 mg/kg; F[1, 12] = 7.84, *p* < 0.01; Fig. 2a) non-cumulatively at 90 min and 240 min, as well as when calculated for the full 4-h time period, while treatment did not affect water intake (F[1, 12] = 0.23, n.s.; Fig. 2b).

### SR-8993 suppresses operant alcohol self-administration, decreases motivation to work for alcohol, and prevents cue- as well as stress-induced relapse (Fig. 3)

**Fig. 3:**
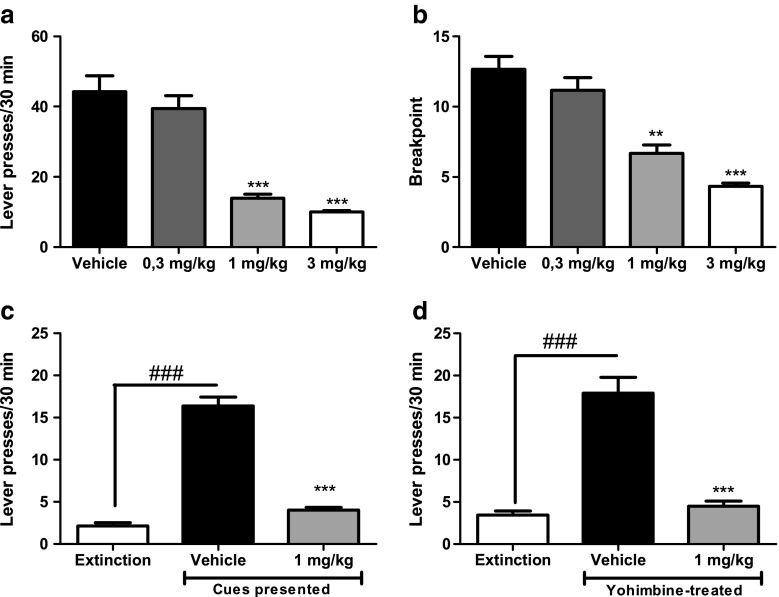
SR-8993 suppresses alcohol self-administration (**a**), decreases motivation to work for alcohol (**b**), and prevents both cue- (**c**) and stress-induced relapse-like behavior (**d**). **a** Operant self-administration of alcohol (10 % *v*/*v*) was significantly attenuated by pre-treatment with 1 and 3 mg/kg SR-8993. **b** Pre-treatment with SR-8993, significantly decreased motivation to work for alcohol delivery as measured using progressive-ratio responding. Breakpoint is defined as the highest number of lever presses the animals performed to receive one dose of alcohol in the drinking cup. **c** Exposure to alcohol-associated cues (light/orange odor) induced reinstatement of lever pressing at the lever previously paired with alcohol delivery (extinction vs. vehicle bars). Pre-treatment with SR-8993 (1 mg/kg) completely blocked this cue-induced reinstatement (vehicle vs. 1.0 mg/kg bars). **d** Similar to what was observed for cue-induced reinstatement, yohimbine-induced reinstatement was blocked by pre-treatment with SR-8993. All values are given as mean ± SEM. ** *p* < 0.01 vs. vehicle, *** *p* < 0.001 vs. vehicle

Operant self-administration was significantly suppressed by pre-treatment with SR-8993 in a dose-dependent manner (F[3, 40] = 34.24; *p* < 0.0001; Fig. 3a) while not significantly affecting inactive lever pressing (F[3, 40] = 0.4; *p* = 0.97; data not shown).

Progressive-ratio break points were significantly suppressed by SR-8993 at both 1 and 3 mg/kg (F[3, 44] = 29.17, *p* < 0.001; post hoc 1 mg/kg *p* < 0.001, 3 mg/kg *p* < 0.001 vs. vehicle; Fig. 3b).

A robust reinstatement of responding on the alcohol-associated lever was found when presenting alcohol-paired stimuli after extinction (reinstatement session vs. last day of extinction: (F[1,18] = 27.4, *P* < 0.001). SR-8993 (1 mg/kg) strongly suppressed cue-induced reinstatement (main treatment effect (F[1,18] = 29.4, *p* < 0.001; Fig. 3C)). Similarly, the pharmacological stressor yohimbine resulted in a robust reinstatement of alcohol seeking (vehicle-treated group/reinstatement vs. last day of extinction (F[1,22] = 11.4, *p* < 0.01). Administration of SR-8993 (1 mg/kg) strongly suppressed stress-induced reinstatement (main treatment effect, F (1, 22) = 8.3, *p* < 0.01; Fig. 3D). Response rates on the inactive lever were unaffected in both paradigms (data not shown).

### SR-8993 reduces escalated alcohol consumption in the intermittent access model and suppresses operant self-administration in animals with a history of escalation (Fig. 4)

**Fig. 4:**
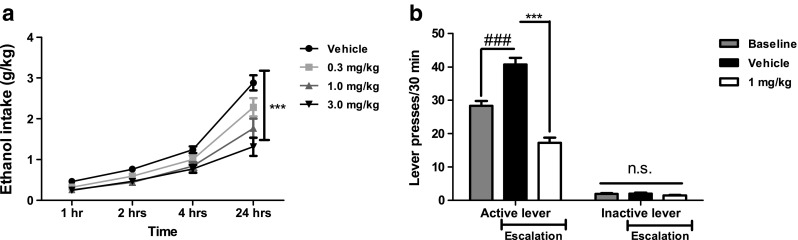
Effects of SR-8993 on alcohol intake (**a**) and operant responding (**b**) in animals with escalated alcohol consumption. Animals were allowed intermittent (24 h, three times per week) access to 20 % alcohol for 15 weeks. **a** During the intermittent access to 20 % alcohol, SR-8993 at 1 and 3 mg/kg significantly attenuated consumption over the 24 h time period. **b** In operant self-administration, a significant escalation of responding from baseline prior to intermittent access to re-training after the intermittent access paradigm was seen (baseline vs. vehicle bars). Pre-treatment with SR-8993 (1 mg/kg) significantly attenuated responding. Baseline indicates responding prior to 15 weeks of intermittent access to 20 % alcohol. The line indicating “Escalation” points to responding after intermittent access following either vehicle or SR-8993 (1 mg/kg) treatment. All values are given as mean ± SEM. **a** *** *p* < 0.001 vs. vehicle, **b** ### *p* < 0.001 vs. extinction, *** *p* = 0.01 vs. vehicle

As previously reported (Simms et al. 2008), intermittent access to 20 % alcohol resulted in escalation of alcohol intake, reaching an average of 2.9 ± 0.2 g/kg/24 h in the vehicle-treated group. SR-8993 at 1 and 3 mg/kg suppressed overall alcohol intake over 24 h but not at any earlier timepoint (Fig. 4a**;** at 24 h: F[3, 28] = 48.3; *p* < 0.001; Newman–Keul post hoc test, vehicle vs. 0.3 mg/kg—n.s.; vehicle vs. 1 mg/kg—*p* < 0.001; vehicle vs. 3 mg/kg—*p* < 0.001).

A robust escalation of lever pressing for 20 % alcohol was seen compared to baseline responding prior to intermittent access exposure (*t* = 20.3, df = 22, *p* < 0.001; Fig. 4b). SR-8993 significantly suppressed lever pressing for 20 % alcohol (*t* = 26.77, df = 22, *p* < 0.001; Fig. 4b) while not affecting activity at the inactive lever. There was no effect of treatment during intermittent access on the treatment effect seen during self-administration (interaction treatment IA × treatment SA, F[2,21] = 0.10; *p* = 0.93); therefore, analyses of baseline vs. escalated intake (“vehicle”) and treatment effect were analyzed separately.

### Effects of SR-8993 on alcohol-related behaviors are behaviorally specific (Table 1)

General locomotor activity was reduced for the highest dose tested (F[2, 21 = 4.19; *p* < 0.05]; post hoc, 3 mg/kg; *p* < 0.01 vs. vehicle) but not for the lower 1 mg/kg dose at which effects on alcohol-related behaviors were observed.

**Table 1:** Control measures evaluating the functional specificity of SR-8993 to anxiety- and alcohol intake-related measures

Measure	Vehicle	1.0 mg/kg	3.0 mg/kg
Locomotion (beam-breaks per 60 min, *n*)	1677 ± 245	1514 ± 233	1011 ± 103**
Lever presses (0.1 % saccharin, 30-min session)	125 ± 10	115 ± 17	n/a
LORR (sleep time, min)	188 ± 5	180 ± 10 (ns)	n/a
BAC at waking (mg/dl)	232 ± 21	216 ± 19 (ns)	n/a
Taste adulteration (intake in ml)	4.1 ± 0.5	3.9 ± 0.6	n/a
Water intake during taste adulteration (intake in ml)	19.3 ± 3.1	18.8 ± 2.6	n/a
Taste adulteration (preference in %)	18.2 ± 1.6	18.4 ± 3.4	n/a
FST (time spent immobile, sec)	173 ± 19	194 ± 18	n/a

SR-8993 did not significantly affect any control measure at the dose active in the alcohol models, 1 mg/kg. The highest dose included in alcohol-related behavioral testing, 3 mg/kg, was shown to have a sedative effect in the locomotor test and was therefore excluded from most of the further evaluations. Behaviors evaluated included, in addition to overall locomotor activity, self-administration of 0.1 % saccharin to check for specificity in treatment for drugs of abuse as well as a locomotor control within the operant self-administration paradigm, sensitivity to the pharmacological effects of alcohol as measured by sleep time in the loss of righting reflex paradigm during and following which effects on blood alcohol concentrations were measured to evaluate alcohol elimination rates. To exclude treatment effects due to alterations in taste perception following treatment with SR-8993, a quinine/saccharin adulteration test was performed and, finally, any effects on depressive-like behavior were evaluated using the forced swim test. All values are given as mean ± SEM

**p < 0.01 vs. vehicle

Operant responding for a non-caloric reward, 0.1 % saccharin was not affected by pre-treatment with 1 mg/kg SR-8993.

Neither the sensitivity to the sedative effects of alcohol (LORR) nor elimination rate of alcohol (BAC) was affected by treatment with SR-8993 (1 mg/kg).

Taste preference was examined as measured by intake of a quinine/saccharin solution. SR-8993 did not significantly affect either water (F (1,11) = 0.5; n.s.) or saccharin/quinine solution intake (F (1,11) = 0.7; n.s.). Most importantly, preference, expressed as the saccharin/quinine solution consumed as percentage of total fluid intake, was unaffected (F (1,11) = 0.3; n.s.).

Depression- and anxiety- like behaviors are related, and we therefore evaluated depression-like behavior in the forced swim test. SR-8993 at 1 mg/kg did not significantly affect time spent immobile in the FST.

## Discussion

The novel, brain-penetrant small-molecule NOP agonist, SR-8993, here showed potential utility in treatment of alcohol use disorders. Systemic administration of SR-8993 significantly suppressed both free-choice limited access drinking and operant alcohol self-administration, as well as progressive-ratio responding, indicating a decreased motivation to obtain alcohol. Effects of SR-8993 on anxiety-like behavior were modest in the absence of prior alcohol exposure, but the pronounced anxiety-like behavior observed during withdrawal from a single large alcohol dose (“hangover anxiety”) was fully reversed by SR-8993. The inverted U-shaped dose-response curve seen in the non-challenged EPM may reflect sedative effects at higher doses. In relation to the reversal of hangover anxiety, escalated alcohol consumption induced by prolonged intermittent access was suppressed by the agonist. SR-8993 further reduced relapse-like behavior, with an equal efficacy to suppress both stress- and cue-induced reinstatement of alcohol seeking. Furthermore, the effective dose of SR-8993 (1 mg/kg) did not influence a wide range of control behaviors, including locomotor activity, operant self-administration of a non-caloric reward (0.1 % saccharin), and sensitivity to the pharmacological effects of alcohol using LORR, as well as elimination of alcohol. However, at a higher dose (3 mg/kg), a mild sedative effect could be seen, indicating the possibility of a narrow therapeutic window for SR-8993 in the models tested.

Early stages of alcohol addiction are considered primarily driven by positively reinforcing alcohol effects and craving for “reward”, while later stages recruit systems promoting negative affective states and alcohol use for negative reinforcing properties, “relief” (Heilig et al. 2010b; Koob and Volkow 2010). N/OFQ and NOP are expressed within areas such as the nucleus accumbens, the ventral tegmental area, and the lateral hypothalamus, and the expression is modulated by exposure to drugs such as cocaine (Caputi et al. 2014; Romualdi et al. 2007) suggesting that the N/OFQ–NOP system is involved in the regulation of reward (Neal et al. 1999a; Neal et al. 1999b). Indeed, intracranial administration of N/OFQ suppresses drug-induced and basal dopamine release in the nucleus accumbens and blocks the reinforcing properties of alcohol (Kuzmin et al. 2003; Martin-Fardon et al. 2000). Here, we show that treatment with the NOP agonist, SR-8993, significantly reduced reward-related alcohol intake in non-operant as well as operant paradigms and that it reduced responding in the progressive-ratio model, indicating reduced motivation to work for alcohol.

Most recent studies evaluating the effects of NOP-agonism have focused on its role in negatively reinforced alcohol consumption, in models of enhanced stress sensitivity such as genetically selected msP rats or Wistar rats with a history of alcohol exposure (Ciccocioppo et al. 2004; Ciccocioppo et al. 2003; Ciccocioppo et al. 2014; de Guglielmo et al. 2014; Economidou et al. 2006). To our knowledge, only one prior study has evaluated the effects of a small-molecule NOP agonist on primary, positive reinforcement by alcohol in non-selected, non-dependent rats. In this study, Kuzmin and colleagues reported a behaviorally selective suppression of alcohol self-administration using Ro 64-6198 (Kuzmin et al. 2007). Our results are consistent with and expand on these findings.

Negatively reinforcing properties of alcohol, together with the escalation of voluntary alcohol consumption, emerge as a result of neuroadaptations following a history of brain alcohol exposure (Heilig et al. 2010a). As mentioned in the “Introduction” section, Wistar rats without a history of dependence are not sensitive to suppression of alcohol self-administration or stress responses by N/OFQ. However, following a history of dependence, administration of N/OFQ has been shown to reduce alcohol intake and produces anxiolytic-like effects (Martin-Fardon et al. 2010). Several approaches have been used to model escalation of alcohol intake and accompanying traits such as increased stress sensitivity, including experimenter-imposed alcohol dependence (Meinhardt and Sommer 2015) or intermittent access to alcohol (Simms et al. 2008). Here, we evaluated the effect of SR-8993 on escalation emerging during intermittent access to 20 % alcohol and on escalated operant alcohol self-administration observed in animals previously exposed to intermittent access. SR-8993 markedly suppressed consumption during intermittent access and reversed the subsequent escalated responding in operant self-administration. We have previously found that activity to reverse “hangover anxiety”, a short term model that lends itself to drug screening, is predictive of activity in models of long term neuroadaptations induced by alcohol (Gehlert et al. 2007; Heilig et al. 2010a). Our findings with SR-8993 in the present study are in line with those observations. Specifically, SR-8993 showed only the modest anxiolytic-like activity in the absence of alcohol exposure but showed high efficacy during withdrawal from a high dose of alcohol and reversed the pronounced anxiogenic-like behavior observed. A history of alcohol exposure has been shown to modulate expression of N/OFQ (Martin-Fardon et al. 2010), indicating neuroadaptations in the system due to a history of alcohol exposure. Of note, SR-8993 has recently shown activity in another condition characterized by sensitized stress systems, an animal model of PTSD (Andero et al. 2013).

Finally, relapse is a key element of addictive disorders. Reinstatement of operant responding following extinction has been widely adopted as an animal model of relapse-like behavior (Bossert et al. 2013; Epstein et al. 2006; Le and Shaham 2002; Shaham et al. 2003). Similar to what is observed clinically, relapse-like behavior is here triggered both by drug-associated cues and by exposure to stressors, including the pharmacological stressor yohimbine. Yohimbine, while not as widely used as foot-shock stress, is a pharmacologically validated model of stress-induced reinstatement (for a review on relapse models see Martin-Fardon and Weiss (2013)) and has been shown to reinstate drug seeking for not only alcohol but also other drugs of abuse (Banna et al. 2010; Buffalari et al. 2012; Shepard et al. 2004). Stress-induced reinstatement is in part driven by endogenous corticotropin-releasing factor (CRF), and both foot-shock stress- and yohimbine-induced relapses to alcohol seeking is blocked by CRF antagonists (Le et al. 2002; Liu and Weiss 2002; Marinelli et al. 2007a). However, it should be noted that recent data suggest the actions of yohimbine on operant responding not to be primarily due to its stress-like effects on motivation for drug seeking. Chen et al. (2015) presented data showing that the dose commonly used for stress-induced reinstatement for alcohol and other drugs of abuse does not induced conditioned place aversion on its own (Chen et al. 2015). Furthermore, they show reinstatement to be due to increased cue-reactivity and/or arousal, since increased responding following extinction and yohimbine-induced reinstatement was seen in a group of animals trained using a cue only (and no reward present). While all stressors do not elicit an identical response and yohimbine does induce a significant corticosterone in rodents (Marinelli et al. 2007b; Simms et al. 2011), it may be advisable to use some caution in terminology and label reinstatement following yohimbine as “yohimbine-induced” and not “stress-induced.” NOP-receptor activation within the amygdala complex has been proposed to oppose stress-induced CRF signaling in this structure by opposing CRF ability to facilitate GABAergic transmission in the CeA—an effect enhanced during alcohol withdrawal (Cruz et al. 2012; Schank et al. 2012). This points to a plausible mechanism for blockade of stress-induced reinstatement involving interactions between NOP and CRF signaling. Genetically selected alcohol-preferring msP rats show increased sensitivity to stress-induced reinstatement (Hansson et al. 2006) and have a regional impairment of N/OFQ–NOP signaling within the CeA (Economidou et al. 2008). Indeed, hypofunction of the N/OFQ system may be a factor underlying high alcohol intake in msP rats, indicating that NOP-agonism may be a viable target in high-intake states. Here, we found that SR-8993 blocked yohimbine-induced reinstatement also in non-alcohol-dependent animals, in agreement with the proposed role of N/OFQ–NOP signaling to oppose CRF-driven stress-induced relapse. Again, our findings agree with and expand on the observations of Kuzmin and colleagues, who reported suppressed relapse-like drinking using the alcohol deprivation model (Kuzmin et al. 2007).

We note that in our present study, SR-8993 showed an unusually broad activity to block relapse-like behavior, in that it blocked reinstatement induced both by the pharmacological stressor and by alcohol-associated cues. Prior work has suggested that these behaviors are mediated by dissociable mechanisms (Liu and Weiss 2002). The broad activity profile of SR-8993 therefore suggests actions on both sets of mechanisms. This is consistent with prior findings obtained with N/OFQ itself (Ciccocioppo et al. 2004; Martin-Fardon et al. 2000) and parallels the observation of activity on both positively and negatively reinforced alcohol self-administration obtained here. The specific effects of different NOP agonists on reward- and stress-related behaviors may be due to differences in subsequent intracellular mechanisms following binding of the agonist to the receptor (Asth et al. 2016). This may be a possible explanation for the range of effects of SR-8993 but requires further evaluation.

In summary, we provide evidence that systemic treatment with the novel, selective, and brain-penetrant NOP agonist SR-8993 has a highly specific and unusually broad in vivo activity suggesting its potential utility as a therapeutic.
